# The role of gut microbiome in colorectal cancer development: a comprehensive analysis based on metabolomics and immunomodulatory mechanisms

**DOI:** 10.3389/fonc.2025.1629495

**Published:** 2025-07-11

**Authors:** Yichi Xu, Bo Wen, Shu Liu

**Affiliations:** Geriatric Department of the First Affiliated Hospital of China Medical University, Shenyang, Liaoning, China

**Keywords:** colorectal cancer, gut microbiome, immunomodulation, carcinogenesis, Wnt/β -catenin signaling

## Abstract

With shifts in lifestyle and dietary habits, the incidence of colorectal cancer has been rising annually, with an increasing prevalence among younger populations, thereby imposing a significant burden on global health. Although the rate of early diagnosis for colorectal cancer has improved due to the widespread use of gastrointestinal endoscopy, many patients still do not experience substantial improvements in survival rates or quality of life. Consequently, there is a pressing need for further in-depth research into the pathogenesis of colorectal cancer, as well as the exploration of potential methods for early diagnosis and precise treatment. As research into the gut microbiome system advances, its remarkable efficacy in the early diagnosis, treatment, and prognosis assessment of various diseases has garnered considerable attention. Variations in the gut microbiome among individuals may result in differential immune responses to specific pathogens or treatment modalities. This article reviews the interaction mechanisms between gut microbiota and immune cells in colorectal cancer, integrating the latest research findings in the field, with the aim of providing potential directions and theoretical foundations for the development of personalized immunotherapy.

## 1 Introduction

The gut microbiome represents a complex and diverse ecosystem that exerts significant influence on human health and the development of diseases (1). Recent advancements in microbiome research have employed multi-omics approaches, including genomic sequencing, transcriptomics, proteomics, metabolomics, and stable isotope probing (SIP), to comprehensively analyze the taxonomic composition, physiological functions, and ecological roles of the gut microbiota (2). Emerging evidence underscores the critical and indispensable role of the gut microbiota in the pathogenesis and progression of various digestive system diseases and disorders. This study focuses on immune regulation to explore the contributions of intestinal microbiota to the initiation and progression of colorectal cancer (CRC), with potential implications for biomarker discovery and the development of targeted therapies.

## 2 Architectural framework and metabolic significance of the gut microbiota

### 2.1 Diversity and stability of the normal gut microbiota

The human gastrointestinal tract serves as the habitat for a diverse array of microorganisms, collectively referred to as the ‘gut microbiota.’ In a typical adult, the gut microbiota encompasses a multitude of bacterial, archaeal, fungal, and viral species that coexist. Through mechanisms such as interspecies competition, metabolic interactions, and host immune regulation, the microbiota sustains normal gut function and enhances resistance to external perturbations. The gut microbiota exhibits considerable inter-individual variation among hosts and demonstrates distinct anatomical distribution patterns. This diversity underscores the stability of the gut microecology and its capacity to resist colonization by external pathogenic bacteria. Simultaneously, the mucosal barrier serves as a physical constraint against bacterial over-colonization. Alterations in dietary composition may induce dysbiosis, which, in turn, can influence environmental homeostasis in humans via metabolite signaling pathways, such as the activation of peroxisome proliferator-activated receptors by butyric acid. Host immune components, including Immunoglobulin A (IgA) and regulatory T cells(Tregs), interact with microbial sensing mechanisms and circadian rhythms to maintain a dynamic equilibrium (3).

Although low microbial diversity in the gut does not inherently signify the presence of disease, it does render the gut more vulnerable to influences from diet, environmental factors, or disease. Such susceptibility may manifest in symptoms like water retention or diarrhea, particularly when associated with a suboptimal diet. Disruptions in the diversity and stability of gut microbiota can compromise the integrity of the gut barrier, resulting in heightened intestinal permeability. This increased permeability may, in turn, initiate inflammatory and immune responses, potentially culminating in the onset of disease.

### 2.2 Functions of the gut microbiota

These gut microorganisms possess substantial metabolic potential and are integral to nutrient absorption, vitamin synthesis, and the regulation of inflammation and immune responses, thereby significantly influencing the host’s physiological functions (4–7). For example, predominant bacterial taxa, including Bacteroides and Firmicutes, are involved in the degradation of complex polysaccharides, leading to the production of short-chain fatty acids (SCFAs). Among these, butyrate is particularly crucial for the energy metabolism of colonocytes. Moreover, evidence suggests that this process can trigger intestinal gluconeogenesis (IGN) via a cAMP-dependent pathway. The activation process in question has demonstrable positive effects on the regulation of glucose levels and energy balance within the body (8).

The phyla Mycobacterium, Clostridium, and Aspergillus are capable of synthesizing various vitamins, particularly vitamins K and B, which include biotin, cobalamin, folate, niacin, pantothenic acid, pyridoxine, riboflavin, and thiamine (9). These microorganisms also facilitate lipid metabolism through the hydroxylation of bile acids and can modulate drug metabolism efficiency and toxic responses by producing secondary bile acids and catalyzing the transformation of xenobiotics (10). Furthermore, microbial-host interactions play a crucial role in regulating the expression of tight junction proteins via pattern recognition receptors (TLRs/NLRs), thereby maintaining the integrity of the gut barrier (11–13). Additionally, the metabolite butyrate contributes to the enhancement of gut barrier function by inhibiting the histone deacetylase mechanism (14, 15).

Research has demonstrated that butyrate exerts distinct regulatory effects on inflammatory responses depending on its concentration. At lower concentrations, butyrate displays anti-inflammatory properties, potentially through the activation of the peroxisome proliferator-activated receptor-γ (PPAR-γ) signaling pathway, inhibition of the nuclear factor kappa-light-chain-enhancer of activated B cells (NF-κB) signaling axis, and subsequent suppression of pro-inflammatory cytokines such as tumor necrosis factor-α (TNF-α) (7, 16). Additionally, butyrate is thought to mitigate inflammatory responses by upregulating the anti-inflammatory cytokine interleukin-10 (IL-10) (17). Conversely, at higher concentrations, butyrate not only fails to inhibit the production of pro-inflammatory factors but may also enhance apoptosis and the release of pro-inflammatory cytokines such as interleukin-1β (IL-1β). This pro-inflammatory effect may involve the activation of G protein-coupled receptors, the lipid transport protein CD36, and the kinase SRC. The findings suggest that butyrate exerts complex and multifaceted regulatory effects on inflammatory responses across varying concentrations. This biphasic effect underscores the importance of considering the dose-dependent impact of metabolites when implementing targeted microbiota interventions.

Meanwhile, gut microbes (e.g. Faecalibacterium prausnitzii) can maintain immune homeostasis by regulating Treg cell differentiation and Th17/Treg homeostasis. Butyrate contributes to the stabilization of Tregs by preserving a low methylation status in the Treg-specific demethylated region (TSDR) of the Foxp3 locus (18). This reduced methylation status is indicative of Treg cell stability and functionality, facilitating the consistent expression of Foxp3 (19). Furthermore, butyrate enhances the suppressive capabilities of Tregs by modulating gene expression at the epigenetic level through the inhibition of histone deacetylases (HDACs) (20). Nonetheless, certain components of the cell wall from butyrate-producing bacteria may counteract this beneficial effect by activating the NOD2 pathway, which recognizes bacterial cell wall components and initiates an immune response. Consequently, in the development of butyrate-based therapeutic strategies, it is imperative to screen for “pure” metabolites devoid of interfering components to prevent unwarranted immune activation and to ensure the efficacy and safety of these interventions in modulating immune responses (20).

Research indicates that dysbiosis of the gut microbiota is intricately associated with the susceptibility to and progression of CRC. Notably, specific gut microorganisms can influence CRC susceptibility and progression by modulating inflammatory responses and DNA damage mechanisms. Furthermore, metabolites produced by the gut microbiota, such as SCFAs with a particular emphasis on butyrate, have been demonstrated to regulate gene expression through the inhibition of HDACs, thereby inhibiting tumor cell proliferation and promoting apoptosis (21). This article will explore this topic in greater detail in subsequent sections.

### 2.3 Characteristics of the gut microbiota in CRC patients

The composition of the gut microbiome exhibits significant differences between individuals with CRC and healthy controls. In CRC patients, the gut microbiota promotes the proliferation of carcinogenic bacteria while reducing populations of probiotic bacteria. Additionally, certain bacterial species may assume distinct roles depending on the stage or anatomical location (proximal or distal) of CRC (22, 23). In a study by Dai et al., seven bacteria enriched in CRC were identified across populations of various nationalities: Bacteroides fragilis, Fusobacterium nucleatum, Porphyromonas acidophilus, Parvimonas micra, Prevotella intermediates, Alistipes finegoldii, and Thermobifida acidaminovorans. Changes in the proportional representation of these microbial species may serve as quantifiable biomarkers for CRC (24). Concurrently, research has demonstrated that Clostridium, Shewanella, and Fusobacterium nucleatum are significantly enriched in the gut microbiota of CRC patients with KRAS mutations. These bacterial communities may contribute to tumor development by promoting inflammation or directly causing DNA damage, such as through colibactin production by pathogenic Escherichia coli. In contrast, in patients with wild-type KRAS, elevated levels of Bifidobacterium and Akkermansia may be associated with anti-inflammatory effects and the inhibition of tumor progression (25). Furthermore, the research conducted by Ma Yanlei’s team at Fudan University on young-onset colorectal cancer (yCRC) demonstrated an increase in gut microbiota diversity among yCRC patients, characterized by distinct metabolic features predominantly associated with DNA binding and RNA-dependent DNA biosynthesis processes (26). Contemporary studies suggest that various factors, such as physiological status, pathological conditions, and environmental exposures, can influence the structural and metabolic composition of gut microbiota in individuals with CRC.

In addition to the alterations in enriched bacterial species observed in CRC patients, certain bacteria demonstrate notable spatial distribution characteristics. Spatial transcriptomics data indicate that Fusobacterium nucleatum is significantly more prevalent at the tumor-infiltrating front compared to the core area, which may be associated with its capacity to facilitate tumor progression and immune evasion (27). The Fap2 protein of Fusobacterium nucleatum is capable of directly binding to the TIGIT receptor on the surface of tumor cells, thereby suppressing the activity of natural killer (NK) cells. This binding mechanism enables tumor cells to evade immune system attacks, thereby promoting tumor growth and metastasis (28). Additionally, Fusobacterium nucleatum can enhance tumor enrichment and progression through the binding of its Fap2 protein to Gal-GalNAc on the surface of tumor cells (29).The diverse mechanisms by which Fusobacterium nucleatum operates within the tumor microenvironment offer new insights into its potential as a therapeutic target.

## 3 Impact of the gut microbiota on the local immune system

### 3.1 Modulatory effects of the gut microbiota on immune cells

The regulatory effects of gut microbes on immune cells are multifaceted and complex, and are characterized by significant individual variability. Firstly, the enteric microbiome drives immunocyte ontogeny and functional maturation, thereby augmenting comprehensive immune responses through engagement of intestinal immune networks.

Secondly, the enteric microbiome also governs immune cell abundance and functional status (30). For instance, gut microbiota drives the development of Tregs and Th17 cells that work in concert to prevent immune overactivation, suppress excessive inflammation, and maintain immune equilibrium. The interaction between gut microbiota and intestinal epithelial cells plays a pivotal role in immune regulation. Intestinal epithelial cells are capable of acquiring antigens from symbiotic bacteria via endocytosis, which facilitates the maintenance of mucosal T cell homeostasis (31). Furthermore, gut microbiota can activate innate immune signaling pathways in epithelial cells through direct contact, thereby modulating the immune response within the gut (32). The gut microbiota plays a crucial role in modulating immune responses by influencing the function of intestinal innate lymphoid cells (ILCs). Research has demonstrated that a specific regulatory subset of ILCs in the gut can inhibit the activation of other ILCs through the secretion of IL-10, thereby safeguarding the gut from inflammatory damage (33).Additionally, the gut microbiota impacts immune responses by modulating the function of antigen-presenting cells (APCs).Evidence suggests that the gut microbiota can restrict the expansion of Th1 cells and facilitate the generation of Treg cells through interactions with CX3CR1+ antigen-presenting cells, thereby maintaining immune homeostasis within the gut (34). Furthermore, gut microbiota modulates the activity of innate immune cells including macrophages and NK cells, consequently augmenting the immune system’s regulatory potential. Nonetheless, the interactions between these regulatory pathways and their spatiotemporal dynamics warrant further comprehensive investigation.

In addition to the aforementioned direct regulatory effects, gut microbial metabolites exhibit significant immunomodulatory properties (3, 9, 21). These metabolites, which encompass SCFAs, bile acids, and vitamins, possess the ability to translocate from the intestinal lumen into the mucosal lamina propria of the intestine. Following gut colonization, these metabolites reprogram host immunological gene networks, thereby influencing downstream effects on immune cell physiology. Disruptions in microbial ecosystems are associated with dysfunctional immune effector cells, which are unable to mount effective antimicrobial defenses, thus increasing susceptibility to infections. Moreover, immune-related disorders, including autoimmune and allergic diseases, may be triggered as a consequence (35).

### 3.2 Effects of gut microbiota on the immune microenvironment and its relationship with CRC

The regulatory effects of the gut microbiota on the immune microenvironment are intricate and exhibit individual variability. Interactions between gut microbes and immune cells, whether direct or indirect, involve complex signaling networks. It has been established that the gut microbiota influences immune cell signaling pathways through pattern recognition receptors, recognition of microbial components, and the release of specific signaling molecules. These substances have been shown to activate or inhibit particular receptors on immune cells, thereby modulating their proliferation, differentiation, and physiological activities, ultimately impacting the immune microenvironment (14, 15, 36, 37). This regulatory influence extends beyond the local immune milieu of the gut, potentially affecting systemic immune status via the gut-liver axis and the gut-brain axis. Furthermore, individual variations in gut microbiota composition may result in differential immune responses to specific pathogens or therapeutic interventions.

Research has established a demonstrable correlation between CRC and the gut microbiota. Specific gut microorganisms, such as Clostridium perfringens and Escherichia coli, have been shown to enhance the growth and invasiveness of colorectal cancer cells (38–40). Conversely, other microorganisms, including Lactobacillus and Bifidobacterium, have been found to inhibit tumor growth (41, 42). Additionally, the gut microbiota may play an indirect role in the immune evasion mechanisms of CRC by affecting the infiltration and activation status of immune cells (43).

## 4 Mechanisms of gut microbiota in the development and progression of CRC

### 4.1 Inflammatory responses triggered by the gut microbiota and the tumor microenvironment

Extensive experimental evidence has substantiated the bidirectional crosstalk between intestinal microbial communities and the neoplastic niche, encompassing both homeostatic immune conditions and therapeutic contexts, such as cytotoxic treatment and immune checkpoint modulation. Dysregulation of the gut microbiota has been shown to contribute directly or indirectly to the progression of CRC by activating pro-inflammatory signaling pathways, inducing chronic inflammation, and causing immune cell dysfunction. For example, the microbial adhesin FadA from Clostridium perfringens interacts with host epithelial receptors, thereby triggering the Wnt/β-catenin and NF-κB signaling pathways. This interaction stimulates the release of inflammatory mediators, including interleukin-6 (IL-6), interleukin-17 (IL-17), and TNF-α, which foster a pro-tumorigenic microenvironment that enhances cancer cell proliferation and metastatic potential (44, 45). Additionally, Bacteroides fragilis toxin (BFT) secreted by B. fragilis has been demonstrated to disrupt the epithelial barrier, resulting in aberrant activation of β-catenin signaling.

Moreover, these toxins have been documented to initiate an inflammatory response via the TLR4/NF-κB signaling pathway, thereby facilitating DNA damage and carcinogenesis (44, 46). Colibactin, a virulence factor produced by Escherichia coli (pks+ E. coli), has been shown to induce DNA double-strand breaks and mutations, which subsequently activate inflammation-related repair mechanisms and expedite tumor development (45). Within a chronic inflammatory milieu, excessive production of reactive oxygen species (ROS) and reactive nitrogen species(RNS) has been observed to cause oxidative DNA damage and the accumulation of mutations. This process has been demonstrated to involve the inactivation of the p53 gene, thereby promoting the malignant transformation of cells. Additionally, the polarization of macrophages towards a pro-tumorigenic M2 phenotype has been reported to result in the secretion of chemokines, such as CCL2, which recruit Th17 cells and establish a pro-tumorigenic, immune-suppressive network (44, 46, 47). Furthermore, the consumption of high-fat diets (HFDs) has been shown to induce gut microbiota dysbiosis, leading to elevated levels of saturated fatty acids (SFAs).

### 4.2 The impact of gut microbiota metabolites on local immune tolerance and their role in regulating tumor immune evasion

The significance of SCFAs as metabolites is noteworthy, with their composition primarily consisting of butyrate, acetate, and propionate. In a healthy physiological state, research has demonstrated that butyrate plays a crucial role in facilitating the differentiation of regulatory T (Treg) cells and maintaining immune homeostasis by inhibiting histone deacetylase (HDAC). In contrast, reduced levels of butyrate in CRC have been shown to impair immune surveillance and promote tumor evasion (45, 46). Acetate and propionate, on the other hand, have been observed to indirectly influence T cell activation, modulating excessive inflammatory responses by regulating the antigen presentation function of dendritic cells (DCs). Furthermore, certain metabolites, such as the secondary bile acid deoxycholic acid (DCA), are produced through the metabolism of Clostridium species. For instance, Clostridium scindens has been found to decrease intracellular calcium ion concentrations in CD8+ T cells by binding to the plasma membrane calcium ATPase (PMCA), thereby inhibiting the nuclear factor of activated T-cells (NFAT) signaling pathway and diminishing the cytotoxic function of these cells. Moreover, research has shown that the activation of the farnesol X receptor (FXR) facilitates the proliferation of Treg cells while inhibiting the differentiation of Th17 cells. This mechanism contributes to tumor immune evasion and the development of an immunosuppressive microenvironment (48). Lipopolysaccharides (LPS) released by Gram-negative bacteria activate myeloid-derived suppressor cells (MDSCs) via the Toll-like receptor 4 (TLR4) pathway, thereby suppressing CD8+ T cell functionality and inducing programmed death-ligand 1 (PD-L1) expression, which in turn promotes immune escape (43, 45). Additionally, succinic acid, a metabolite of Clostridium nucleatum, has been found to inhibit the cyclic GMP-AMP synthase-interferon beta (cGAS-IFN-β) pathway and decrease Th1 chemokine secretion through the activation of the succinate receptor 1-hypoxia-inducible factor 1-alpha-enhancer of zeste homolog 2 (SUNCR1-HIF-1α-EZH2) axis in tumor cells. This process further diminishes CD8+ T-cell infiltration and function, leading to resistance to immunotherapy (49). Furthermore, it has been demonstrated that probiotic metabolites, such as indole propionic acid (IPA), can enhance the efficacy of anti-programmed cell death protein 1 (anti-PD-1) therapy through the activation of the aryl hydrocarbon receptor (AhR). This finding suggests that the specific metabolites derived from targeted flora possess the capability to alter the immunosuppressive microenvironment (50). In conclusion, it has been demonstrated that the gut microbiota can modulate the immune microenvironment via metabolite production. Nonetheless, the nature of the metabolites and the existing microenvironmental conditions are critical in determining whether this modulation is pro-carcinogenic or anti-carcinogenic.

## 5 Influence of the gut microbiota on colorectal cancer-associated signaling pathways

### 5.1 Wnt/β-catenin signaling pathways

Aberrant activation of the Wnt/β-catenin signaling pathway constitutes a pivotal mechanism in the pathogenesis of CRC. This pathway’s regulation is influenced not only by genetic mutations, such as the inactivation of the APC gene, but also by the dynamic equilibrium of the intestinal microbiota. Clostridium perfringens adhesin FadA has been shown to activate the Wnt/β-catenin signaling pathway upon binding to host epithelial cells, leading to increased expression of oncogenic and inflammatory responses (44, 48, 51). Research conducted by the Institute of Microbiology, Chinese Academy of Sciences, indicates that ARNT transcription factors modulate Wnt/β-catenin signaling by regulating neutrophil recruitment and activity, as well as influencing the composition of the gut microbiota. A deficiency in ARNT exacerbates the formation of neutrophil extracellular traps (NETs) and the release of inflammatory cytokines, thereby deteriorating the tumor microenvironment (52). The research team, led by Yuhao Wang at Zhejiang University, has identified that the over-activation of Wnt signaling is modulated by intestinal microbiota, which suppresses the expression of the long-chain non-coding RNA Snhg9. This suppression subsequently accelerates tumor progression by promoting the dissociation of SIRT1 from p53, thereby inhibiting the activity of the latter. Notably, the up-regulation of Snhg9 expression following antibiotic administration further exacerbates the progression of CRC (47). Conversely, in animal models, the administration of Portulaca oleracea extract (POE) to CRC-afflicted mice led to the identification of 20 distinct microbiota potentially involved in CRC development via the Wnt/β-catenin signaling pathway. Additionally, c-Myc and cytosolic protein D1 were identified as critical downstream targets within the Wnt/β-catenin signaling pathway. *In vitro* experiments further demonstrated that POE could inhibit the proliferation of CRC cells by downregulating the expression of c-Myc and cell cycle protein D1, thereby inactivating the Wnt/β-catenin signaling pathway (53). Simultaneously, Roya et al. employed both *in vivo* (using a CRC mouse model) and *in vitro* methodologies to illustrate that a consortium of probiotic Lactobacillus species (specifically, the potentially probiotic L.C.) effectively suppressed tumor growth by inhibiting the Wnt/β-catenin signaling pathway (54). This finding underscores the dual role of gut microbiota in modulating the Wnt/β-catenin signaling pathway through epigenetic mechanisms.

### 5.2 PI3K/Akt signaling pathway

The PI3K/Akt signaling pathway plays a crucial role in CRC metastasis and drug resistance. Research conducted by Jingyuan Fang and colleagues revealed that the microbial virulence factor RadD from Fusobacterium nucleatum facilitates oncogenic progression by exploiting the CD147 receptor to activate the pro-tumorigenic PI3K-Akt-NF-κB signaling cascade in malignant cells. Additionally, SCFAs, such as butyric acid, have been shown to inhibit tumor progression by suppressing Akt phosphorylation. In contrast, a reduction in SCFA levels, due to microbial dysbiosis, has been associated with the activation of Akt signaling. Experimental animal studies have demonstrated that the administration of butyric acid-producing bacteria, such as Bifidobacterium bifidum, can counteract this effect by restoring SCFA levels (55).

### 5.3 NF-κB signaling pathway

The NF-κB signaling pathway has been recognized as a crucial intermediary linking intestinal inflammation to CRC. As discussed in Section 4.1, BFT toxins produced by *C. fragilis* have been demonstrated to initiate inflammatory responses and facilitate DNA damage and carcinogenesis via the TLR4/NF-κB pathway (44, 46). *Clostridium nucleatum* has been documented to secrete pro-inflammatory factors, such as IL-6 and IL-8, through the TLR4/NF-κB axis, thereby intensifying damage to the intestinal epithelium (53). Additionally, neutrophils deficient in ARNT have been shown to augment NET formation and inflammatory responses through CXCR2 signaling, which subsequently activates the NF-κB pathway and promotes tumor growth (51). A study by Yijia Wang et al. proposed that *Fusobacterium*, identified as a driving bacterium of CRC, not only induces colitis but also enhances the expression of NF-κB p65 in the nucleus of intestinal epithelial cells. These molecular alterations contribute to carcinogenic processes and tumor progression (38). According to the findings of Yuhao Wang and colleagues, within a low-level inflammation model, the bacterial population has been shown to inhibit NF-κB activity by sustaining IL-22 secretion. In contrast, the lack of IL-22 has been associated with increased expression of Snhg9, which subsequently facilitates carcinogenesis via the SIRT1-p53 axis (47).

### 5.4 TGF-β signaling pathway

Research has demonstrated that the TGF-β signaling pathway plays a dual role in CRC, exhibiting both tumor-promoting and tumor-suppressing effects. In a healthy state, retinoic acid (RA) has been shown to maintain the stability of the intestinal immune system by promoting the development of Treg cells and the production of IgA by B cells through the TGF-β signaling pathway (56). The team led by Fang Jingyuan discovered that dysbiosis, such as a reduction in Bifidobacteria, activates the urea cycle in the host. The resulting high urea load disrupts intestinal immune homeostasis by inhibiting the binding of p-STAT1 to SAT1 in macrophages, leading to their differentiation into immunosuppressive subtypes (55). Moreover, the current study reveals a positive correlation between the high expression of the CCDC113 gene and the activation of the TGF-β signaling pathway. This finding suggests that the intestinal microbiota may influence the dynamic balance of this pathway by regulating host gene expression (55).

## 6 Role of the gut microbiota in colorectal cancer targeted therapy and immunotherapy

In this section, we have summarized the recent research on gut microbiota in immunotherapy for colorectal cancer for reference (**Table 1**).

**Table 1:** Researches on gut microbiota in immunotherapy for colorectal cancer.

Flora	Immunotherapy type	Sample Type	RESULTS
*Fusobacterium nucleatum*	anti-PD-1	Patient specimens:CRC patients treated with PD-1 blockade therapy and measured F.nucleatum abundance in tumor tissues and feces. Cell: Human CRC cell lines DLD1 and Caco-2,mouse colon cancer cell line CT26. Mice: CT26.WT were implanted into the lateral wings of BALB/c mice, followed by intratumoral injection of F. nucleatum, Escherichia coli DH-5α or PBS,then injected anti-PD-L1 monoclonal antibody (mAb) or an isotype control mAb into the abdominal cavity.	*F.Nucleum* induces PD-L1 expression by activating STING signaling and increases the accretion of interferon-γ (IFN-γ)+CD8+TIL during PD-L1 blockade therapy, thereby enhancing the anti-tumor response to PD-1/PD-L1 checkpoint blockade (57).
*Agathobacter* and *Blautia*	anti-PD-1 combined Anti-EGFR	Patient specimens:patients with ctDNA RAS/BRAF WT and MSS disease.	*Agathobacter* and *Blautia* species are capable of producing SCFAs, which may represent the mechanism through which these bacteria augment the anti-tumor activity of cetuximab and avirumab (58).
MS-20	anti-PD-1	Mice: BALB/c mice were inoculated subcutaneously with live CT-26 cells and intraperitoneally injected with anti-PD1 antibodies. They were then randomly assigned to receive either sterile water or different concentrations of MS-20 orally.	The combination of MS-20 and an anti-PD1 antibody demonstrated a marked efficacy in the inhibition of tumor growth, accompanied by an augmentation in the total CD8+ T cell population and the activation of CD8+ T cells within the tumor microenvironment. This combination also resulted in the inhibition of PD1 expression, the enhancement of the efficacy of anti-PD1 monotherapy, and the promotion of intra-tumour CD8+ T cell infiltration (59).
*B. pseudolongum*	anti-PD-1 and anti-CTLA-4	Mice: MC38 CRC cells were implanted into the lateral wings of GF or SPF mice followed by ICB therapy once tumors were palpable.	The *B.pseudolongum* strain greatly enhances the efficacy of ICB therapy in intestinal and epithelial tumor mouse models by enhancing the CDC-dependent TH1 cell circuit. A2AR signaling may be an important anti-tumor pathway (63).
CTLA-4 inhibitors that lack an Fc domain	anti-CTLA-4	Mice: The WildR mice were colonized with the objective of establishing an ICB-induced colitis model.	CTLA-4 inhibitors lacking the Fc structural domain do not induce colitis in colitis-prone mice when receiving conventional anti-CTLA-4 antibodies. Notably, anti-CTLA-4 VHHs can resist tumor responses without inducing gut irAEs in mice (64).
*Bifidobacterium*	anti-CTLA-4	Mice: Female mice aged between 6 and 14 weeks were subjected to an Il-10 KO procedure and then treated with vancomycin. Following a 14-day period, DSS was added to the mice’s drinking water. Thereafter, the mice were injected with either an anti-CTLA-4 monoclonal antibody or a control.	*Bifidobacterium* exerts a systematic influence on the composition of the gut microbiota, acting in a manner contingent on Treg cells.*B. breve* and *L. rhamnosum* are two functional strains extracted during CTLA-4 blockade to improve intestinal immunity. The IL-10/IL10Ra signaling loop and mitochondrial activity in Tregs have been hypothesized to play a pivotal role in attenuating intestinal inflammation (65).
*Lachnospiraceae* and *Ruminococccaceae*	SCXN	Cell: CT26 murine colon carcinoma cells and CT26-luc Mice: The selection of 4-6 week old female Balb/c and C57BL/6 mice is imperative for the establishment of CT26 or CT26/MC38 tumor models.	The efficient regulation of gut microbiota and enhanced SCFAs generation by SCXN built a tumor-suppressing and beneficial microorganism-supporting intestinal microenvironment (66).
Probio-M9	anti-PD-1	Mice: Mice were randomized into four groups(Model, prophylactic Probio-M9, anti-PD-1 combined treatment group, therapeutic Probio-M9 and anti-PD-1 combined treatment group, medical control group which treated with anti-PD-1 only),and separately given Probio-M9 or normal saline.	Probio-M9 has been demonstrated to enhance tumor suppression in a manner dependent on anti-PD-1, through the generation of advantageous metabolites, the encouragement of CTL infiltration and stimulation, and the curtailment of Treg cell function within the TME (67).
*Faecalibacterium prausnitzii*	anti-PD-1 and anti-CTLA-4	Patient specimens:A comparative analysis of fecal samples from patients receiving ICI treatment, with and without concomitant gastrointestinal toxicity, is to be performed using omics. Mice: Mice were administered 3% DSS and subsequently induced to form an immune checkpoint inhibitor-associated colitis model using dual anti-PD-1 and anti-CTLA-4 antibodies. Then, a quantity of mice is injected with *Faecalibacterium prausnitzii*.	*F.prausnitzii* administration ameliorates ICI-induced colitis, reshapes the gut microbial composition, and enhances the antitumor activity of immunotherapy (68).

### 6.1 Anti-EGFR therapy

The effectiveness of epidermal growth factor receptor (EGFR)-targeted therapies, such as cetuximab, is intricately linked to the composition of the gut microbiota. Research has shown that Fusobacterium nucleatum can induce resistance to EGFR-targeted treatments by activating the NF-κB and Wnt/β-catenin signaling pathways. This mechanism may involve the establishment of a pro-inflammatory microenvironment and the promotion of tumor cell survival pathways (57). Nonetheless, probiotic interventions have been shown to partially mitigate this resistance. Specifically, Agathobacter and Blautia species have been reported to suppress the inflammatory response and enhance drug sensitivity by modulating the composition of the intestinal microbiota and increasing the secretion of SCFAs (58). Moreover, research has demonstrated that the gut microbiota metabolite indolepropionic acid, co-produced by Lactobacillus murinus and Tannerellaceae species, significantly enhances the efficacy of anti-PD-1 monoclonal antibodies. This enhancement is mediated through a mechanism involving increased CD8+ T-cell infiltration and downregulation of PD-1 expression, indicating the synergistic potential of this microbial metabolite with EGFR-targeted therapies (59). Additionally, preclinical models indicate that antibiotic-induced dysbiosis exacerbates the toxicity of EGFR inhibitors, whereas probiotic interventions mitigate intestinal mucosal damage and extend the therapeutic window (60).

### 6.2 Anti-VEGF therapy

The effectiveness of anti-angiogenic agents, such as bevacizumab, is closely linked to metabolites produced by gut microbiota. Recent clinical trials have indicated that propionate, a metabolite derived from Ricardia spp., significantly enhances the anti-angiogenic effects through a synergistic mechanism. This involves promoting apoptosis in tumor cells and facilitating the maturation of dendritic cells in combination with αPD-L1. These findings suggest a promising strategy for treating low-grade rectal cancer using a radioactive hydrogel, 177Lu-RH@SP. Additionally, the integration of fecal microbiota transplantation (FMT) with bevacizumab has shown substantial therapeutic advantages in cases of refractory CRC. Mechanistic investigations have revealed that microbial colonies inhibit tumor angiogenesis by regulating gene expression within the vascular endothelial growth factor (VEGF) pathway, including genes such as MMP2 and HIF-1α (61). Bacteriotherapy, a therapeutic approach developed by Recolony in Switzerland, has further substantiated the clinical potential of interventions involving bacterial flora. This treatment involves the supplementation of butyric acid-producing bacteria, which activate immune cells and enhance anti-tumor angiogenesis. The company has announced its intention to initiate the inaugural human phase Ib clinical study in the latter half of the present year.

### 6.3 Immune checkpoint inhibitor therapy

The influence of intestinal microbiota on immune checkpoint inhibitor (ICI) therapy has emerged as a significant focus of research. Although microsatellite-stable (MSS) CRC typically shows a low response rate to PD-1/PD-L1 inhibitors, interventions targeting the bacterial microbiota have been shown to improve treatment efficacy (62). Notably, the presence of Clostridium nucleatum has been found to enhance the efficacy of PD-L1 blockade under certain conditions. Research conducted by Qin Huanlong’s team revealed that Clostridium perfringens promotes the secretion of interferon-γ (IFN-γ) by activating the STING pathway, thereby augmenting the antitumor activity of CD8+ T cells (57). Additionally, animal studies have provided experimental evidence that Bifidobacterium pseudolongum enhances the recruitment of tumor-infiltrating lymphocytes (TILs) by ICI through the activation of the adenosine A2A receptor by the metabolite inosine (63). Machine learning modeling analyses have demonstrated a positive correlation between the presence of SCFAs-producing microbiota, such as Fusobacterium and Lactobacillus, and the response rates to ICIs. In contrast, a higher abundance of Mycobacterium pseudolongum has been linked to drug resistance (60, 63). A considerable portion of contemporary research is concentrated on CD8+ T cells, as the response to PD-1 is mediated by Tpex cells, which rely on the activation of CD8+ T cell stem cells for differentiation. Nevertheless, CD8+ T cells are not confined to the gut. Modulating the composition of intestinal microbiota can alter the production of their metabolites, potentially disrupting the programming of CD8+ T cells to activate Tpex cells. This disruption may influence the quantity of memory T cells in the peripheral blood, thereby posing new risks to the organism. This represents a notable gap in the current body of research.

The efficacy of immunotherapy is accompanied by certain limitations, notably the occurrence of Immune Checkpoint Inhibitor-Associated Colitis, which has been identified as a significant cause of treatment interruption. In the previous year, researchers from the University of Michigan published a study in *Science* addressing this issue. They transplanted the gut microbiota from field-captured mice into conventional laboratory mice, thereby creating a novel model that replicates human immunotherapy-associated colitis (64). The study demonstrated that the over-activation of IFNγ-producing CD4+ T cells contributes to intestinal inflammation, and that the Fcγ receptor signaling pathway plays a role in the depletion of Treg cells. Based on these findings, the research team developed anti-CTLA-4 nanobodies lacking the Fc structural domain. The successful generation of this novel antibody in a murine model was accomplished with the objective of enhancing anti-tumor immune responses while avoiding the induction of colitis-related side effects. This discovery lays the groundwork for the development of next-generation CTLA-4 inhibitors. In clinical applications, Bifidobacterium bifidum has been shown to provide a protective effect against immunotherapy-associated colitis, offering a potential therapeutic intervention for this condition. It has been demonstrated that this bacterium enhances the immunosuppressive function of Tregs by upregulating the expression of IL-10 receptor alpha (IL-10Ra) and IL-10 in intestinal Treg cells (65). Therefore, prophylactic supplementation with specific probiotics prior to the commencement of immunotherapy may represent a viable strategy to reduce the risk of colitis.

### 6.4 Other treatments

The delivery of therapeutic proteins can be effectively facilitated through the construction of recombinant plasmids, which have been shown to be successful in synthesizing cytotoxic proteins, prodrug-converting enzymes, and proteins involved in angiogenic regulation. The domain of RNA interference (RNAi) is currently a focal point of research, with investigations aimed at developing RNAi molecules. An illustrative example is the capecitabine-containing prebiotic nanoparticle (SCXN), developed by the Shanghai Institute of Pharmaceutical Sciences, Chinese Academy of Sciences. This system employs xylan-stearic acid coupling (Sxy) as the carrier for encapsulating capecitabine within the nanoparticle’s core. Upon oral administration, the nanoparticle undergoes targeted degradation in the intestine, enabling the sustained release of the encapsulated drug. This design confers a dual advantage: it not only prolongs drug clearance and enhances intratumoral accumulation but also utilizes xylan as a prebiotic to elevate the ratio of prebiotics and short-chain fatty acid production. In murine models with a high tumor burden, SCXN has been shown to significantly increase intratumoral drug concentration. In mice with a high tumor burden, SCXN markedly enhanced intratumoral CD8+ T-cell infiltration and increased tumor suppression from 5.29% to 71.78% with the administration of the free drug. This integrated strategy of colony modulation combined with chemotherapy represents a significant advancement in CRC treatment (66). An alternative approach involves reprogramming bacteria by designing metabolic pathways for the synthesis of therapeutic molecules. This metabolic engineering strategy, based on modifications to the bacterial genome, enables the synthesis of therapeutic molecules beyond proteins and eliminates restrictions on the type of therapeutic substances delivered. Further investigation is necessary to determine the extent to which reconstruction impacts the composition and function of the organism’s microenvironment.

## 7 Future perspectives

The gut microbiota influence molecular signaling networks, such as Wnt/β-catenin, PI3K/Akt, and NF-κB, in CRC through complex regulatory mechanisms, thereby playing a crucial role in enhancing the efficacy of targeted therapies and immunotherapies. Future research should aim to further elucidate the specific strains involved and their mechanisms of action, with the goal of developing individualized therapeutic strategies based on microbial colony typing. These strategies might include combinations of FMT, probiotics with targeted pharmaceuticals, or the engineering of microbial strains through synthetic biology to improve therapeutic outcomes. Additionally, strengthening interdisciplinary collaboration holds significant promise for advancing gut microbiota research. The interplay between gut microbiota and the immune microenvironment is inherently interdisciplinary, encompassing fields such as biology, medicine, and microbiology. It is posited that through interdisciplinary cooperation, researchers can collectively address the challenges faced in this domain, facilitate the translation and application of research findings, and thereby contribute more substantially to scientific and medical advancements.

## 8 Conclusion

The investigation of gut microbiota is profoundly reshaping our comprehension and clinical application of immunotherapy for colon cancer. Beginning with the initial observation of the correlation between microbiota composition and treatment response, the field has progressed to an in-depth analysis of molecular mechanisms and the development of innovative intervention strategies, marking a period of rapid translational advancement. Future approaches will likely involve the use of fecal macro-genomic sequencing to determine patients’ intestinal microbiota composition, thereby enabling the creation of tailored microbiota intervention programs. Metabolite levels will be monitored throughout treatment, with the program dynamically adjusted in four stages to ensure a patient-centered therapeutic strategy. Each of these stages will inevitably impose higher demands: precise analysis and administration of personalized microbiota intervention protocols, identification of more accurate and universal metabolites, and the ongoing development of immune checkpoint inhibitors with optimized therapeutic dosages and minimized side effects.
